# Molecular Epidemiology of Adenovirus Type 7 in the United States, 1966–2000^1^

**DOI:** 10.3201/eid0803.010190

**Published:** 2002-03

**Authors:** Dean D. Erdman, Wanhong Xu, Susan I. Gerber, Gregory C. Gray, David Schnurr, Adriana E. Kajon, Larry J. Anderson

**Affiliations:** * Centers for Disease Control and Prevention, Atlanta, Georgia, USA; † Chicago Department of Public Health, Chicago, Illinois, USA; ‡ Naval Health Research Center, San Diego, California, USA; § California Department of Health Services, Berkeley, California, USA; ¶ Lovelace Respiratory Research Institute, Albuquerque, New Mexico, USA

**Keywords:** Adenovirus type 7, restriction enzyme analysis, molecular epidemiology

## Abstract

Genetic variation among 166 isolates of human adenovirus 7 (Ad7) obtained from 1966 to 2000 from the United States and Eastern Ontario, Canada, was determined by genome restriction analysis. Most (65%) isolates were identified as Ad7b. Two genome types previously undocumented in North America were also identified: Ad7d2 (28%), which first appeared in 1993 and was later identified throughout the Midwest and Northeast of the United States and in Canada; and Ad7h (2%), which was identified only in the U.S. Southwest in 1998 and 2000. Since 1996, Ad7d2 has been responsible for several civilian outbreaks of Ad7 disease and was the primary cause of a large outbreak of respiratory illness at a military recruit training center. The appearance of Ad7d2 and Ad7h in North America represents recent introduction of these viruses from previously geographically restricted areas and may herald a shift in predominant genome type circulating in the United States.

Human adenoviruses (Ads) comprise 51 serotypes (*1*); they are ubiquitous and responsible for a wide range of clinical syndromes. Among recognized serotypes, Ad type 7 (Ad7) (and to a lesser extent Ad type 3) is most often associated with severe disease (*2*). Although Ad7 infections typically result in mild upper respiratory tract illnesses and conjunctivitis, infections can also lead to more serious lower respiratory tract illnesses, disseminated disease, and death, particularly in infants and persons with underlying immunologic or respiratory compromise (*3*–*7*). Ad7 infections have also been associated with diseases of the central nervous system (*8*,*9*) and long-term respiratory sequelae that include bronchiectasis and hyperlucent lung or McLeod syndrome (*10*).

Ad7 accounts for nearly 20% of all Ads reported to the World Health Organization (*11*), and family clusters and institutional and communitywide outbreaks of Ad7 disease have been extensively documented (*5*,*12*–*18*). Three types of outbreaks have been described (*12*): i) outbreaks that occur during the winter months among institutionalized infants (<2 years of age) that result in high rates of severe illness and death; ii) periodic nonseasonal communitywide outbreaks involving older children and adults with infrequent serious outcomes; and iii) outbreaks of acute respiratory disease among new military recruits. Outbreaks of acute respiratory disease due primarily to Ad7 and Ad4 were an important cause of illness in new military recruits in the United States until live enteric-coated Ad4 and Ad7 vaccines began to be routinely administered in 1971 (*19*). The recent cessation of production and administration of these vaccines has resulted in a resumption of Ad-associated acute respiratory disease outbreaks at military recruit training centers throughout the United States (*20*–*22*).

To facilitate study of the molecular epidemiology of Ad7, a classification system based on restriction enzyme analysis of Ad genomic DNA was devised by Li and Wadell (*23*) and later revised by Li et al. (*24*). Their system uses *Bam*HI as the "type" defining enzyme, with different genome types denoted with a character, e.g., “p” for the Ad7 prototype strain, Gomen; and then “a” through “k.” Genome types that are further distinguished by restriction pattern with additional selected enzymes are given an Arabic numeral (e.g., Ad7p, p1, a, a1-6). Their system has been widely used to correlate genome types with geographic distribution and pathogenic potential.

Both globally dispersed and geographically restricted genome types of Ad7 have been identified by restriction analysis, and regional shifts or replacements of predominant genome types have been documented on different continents. Among the 3 Ad7 genome types first distinguished by restriction analysis (*25*), two shown to be serologically distinct (*26*) were designated Ad7p (Gomen) and Ad7a (S-1058), and a third, designated Ad7b, was thought to be associated with more severe illness (*12*). Ad7b eventually spread worldwide (*27*–*30*), displacing formerly common genome types (i.e., Ad7p, Ad7a, Ad7a1-6, Ad7c, and others) that are now rarely detected. Exceptions to this pattern have been reported. In the former Soviet Union, a successive shift from Ad7a and Ad7a1-5 to Ad7f1 during 1976-1979 and 1986-1988 was reported (*31*). In South America, a shift from Ad7c to Ad7h occurred in 1986 (*32*), and Ad7h has subsequently caused serious respiratory illness in infants and young children in Chile and Argentina (*33*,*34*). In the early 1980s in China, a new genome type, Ad7d (*27*), replaced Ad7b as the predominant circulating virus. Recent reports suggest that Ad7d and Ad7h have spread beyond their formerly geographically restricted regions. Ad7d was identified in Japan in 1987 (*35*), and countrywide epidemics of Ad7 that began in 1995 in Korea (18; Hoan-Jong Lee, pers. comm.) and Japan (*15*,*36*) were attributed to Ad7d and a closely related genomic variant, Ad7d2, respectively. Ad7d2 has emerged as the predominant strain circulating in Israel since 1992 (*37*). Ad7h was first reported outside South America in 1996, in Japan (*36*,*38*).

Beginning in the fall of 1998, an outbreak of Ad7 infection occurred at a pediatric chronic-care facility in Chicago and subsequently spread to a tertiary-care hospital, where staff from two clinic units were infected (*17*). This multi-site outbreak was associated with considerable illness and death among residents of the chronic-care facility. Isolates from this outbreak were identified by restriction enzyme analysis as Ad7d2. The appearance of this new genome type prompted us to study the temporal and geographic distribution of Ad7 genome types in the United States to better characterize the emergence and spread of this virus.

## Materials and Methods

### Ads

Of 297 Ad field isolates obtained from the Centers for Disease Control and Prevention (CDC) archives, state public health laboratories, university hospitals, and military training centers, 166 confirmed as Ad7 were selected for genome type analysis (Table 1). Of these, 116 were obtained from 1966 to 2000 from civilians in 25 states and eastern Ontario, Canada; 50 were obtained from February 1997 to May 1998 from military recruits attending training centers in five states (*20*). Isolates were selected to achieve broad geographic and temporal distribution. Because detailed demographic, epidemiologic, and clinical data from patients were limited, they were not included in this report. Most civilian isolates were obtained from individual cases or family clusters of Ad7 disease, ranging from mild upper respiratory illness to severe lower respiratory tract illness and death. Where civilian outbreaks of Ad7 illness were recognized, only one representative isolate was included in the 166 sample for analysis. Approximately 10% of Ad7 isolates from military recruits with respiratory illness were sampled from all five training sites and were selected to be evenly spaced over the designated time period. Reference strains Gomen and S-1058 were obtained from the American Type Culture Collection (ATCC, Rockville, MD). Reference isolates of Ad7d2 were obtained from a postmortem rectal swab from a 4-month-old baby in Israel in 1993, and Ad7h was obtained during a regional outbreak of respiratory illness in Chile in 1998. All Ad isolates were passaged at least once in A549 cells before restriction analysis. Type-specificity of all Ad7 field isolates was confirmed by neutralization or Ad7 type-specific polymerase chain reaction assay (PCR) (*39*).

**Table 1:** Human adenovirus 7 (Ad7) field isolates from the United States and Canada, 1966–2000

Location	Isolation year(s)	No. Ad7 isolates
Canada		
Eastern Ontario	1999, 2000	3
United States		
Alabama	1985, 1986	2
Arizona	1995, 1998	3
California^a^	1997	1
California	1981- 4, 1987, 1990, 1992, 1995, 1996	15
Colorado	1987	1
Florida	1986, 1996	2
Georgia	1996	1
Illinois^a^	1996 - 98	28
Illinois	1997	2
Iowa	2000	1
Kansas	1995, 1997	3
Louisiana	1996	2
Maine	1981	1
Maryland	1991, 1993 - 95	16
Massachusetts	1998, 1999	2
Michigan	1986	1
Mississippi	1986	1
Missouri^a^	1997	9
Missouri	1966, 1998, 1999	8
New York	1970, 1985, 1990, 1991, 1993, 1995-97, 1999, 2000	31
Ohio	1993-95, 1997, 1998	10
South Carolina^a^	1997, 1998	11
South Carolina	1998	1
South Dakota	1987	1
Tennessee	1997	2
Texas^a^	1998	1
Texas	1999, 2000	2
Virginia	1985	1
Washington	1996	1
Wisconsin	1996, 1998	3
North America (total)	1966-2000	166

^a^Ad7 isolates obtained from military recruit training centers.

### DNA Restriction Analysis

Ad genomic DNA was extracted by a modification of the method of Deryckere and Burgert (*40*). Briefly, isolates were grown in 75-cm^2^ confluent flasks of A549 cells until the 4+ stage of cytopathic effect was attained. The contents of the flask were centrifuged at low speed to remove cells, and the supernatant was transferred to an ultra-centrifuge tube and centrifuged for 2 hours at 100,000 x *g*. The virus pellet was resuspended in 400 μL of Tris buffer (pH 7.4) with 1% sodium dodecyl sulfate and sequentially digested with DNAse free RNAse A (0.1 mg/mL) and proteinase K (0.5 mg/mL). The digest was extracted twice with equal volumes of phenol and chloroform/isoamylalcohol (24:1) and once with chloroform/isoamylalcohol alone. The purified DNA was then precipitated with absolute ethanol and washed once with 75% ethanol, and the pellet was resuspended in 100 μL of dH_2_O. Enzyme digestions were carried out according to manufacturer's instructions (Boehringer Mannheim Biochemicals, Indianapolis, IN). DNA from all Ad7 isolates was digested with *Bam*HI and *Sma*I, and selected isolates were also digested with enzymes *BcI*I, *Bgl*I, *Bgl*II, *Bst*EII*, Eco*RI, *Hpa*I, *Hin*dIII,*Sal*I, *Xba*I, and *Xho*I. Enzyme digests were electrophoresed at 100 volts for 5 hours on 0.8% agarose gels, and the DNA bands were visualized by ethidium bromide staining. Restriction fragment size(s) was interpolated from DNA molecular weight standards included in each run. Restriction patterns were compared with previously published profiles (*24*,*37*,*41*,*42*), and the identification of genome types followed the denomination system of Li et al. (*24*).

### DNA Sequencing

The hypervariable region of the hexon protein gene corresponding to nucleotides 403 to 1356 (Gomen), which have been shown to encode the residues that define Ad serotype, was PCR amplified from selected Ad7 isolates as described (*43*) and sequenced by using the DyeDeoxy Terminator Cycle Sequencing Kit and ABI 373A automated DNA sequencer (Applied Biosystems, Foster City, CA). Nucleotide sequences were determined for both PCR product strands. Sequence analysis was performed by using the Wisconsin Package ver. 10.0 (Genetics Computer Group, Madison, WI). Hexon gene sequence data for the reference Ad7d2 strain from Israel were submitted to GenBank (accession number AF321311).

## Results

### Ad7 Genome Types Identified

DNA restriction analysis of the 166 Ad7 field isolates identified 108 (65%) as Ad7b, 46 (28%) as Ad7d2, 4 (2%) as Ad7h, 3 (2%) as Ad7p, 3 (2%) as Ad7a, and 2 (1%) as Ad7a3. Restriction profiles of representative Ad7b, Ad7d2, and Ad7h isolates for selected endonucleases are shown in the Figure. All U.S. (and eastern Ontario, Canada) Ad7d2 isolates and an Ad7d2 reference strain from Israel (*37*) gave identical restriction patterns for *Bam*HI, *BcI*I, *Bgl*I, *Bgl*II, *Bst*EII*, Eco*RI, *Hpa*I, *Hin*dIII, *Sal*I, *Sma*I, *Xba*I, and *Xho*I. Identical restriction profiles were also obtained with four U.S. Ad7h isolates and a 1998 isolate of Ad7h from Chile, which were similar to profiles described for Ad7h strains isolated in Argentina and Chile (formerly designated Ad3f) (*41*,*42*,*44*).

**Figure:**
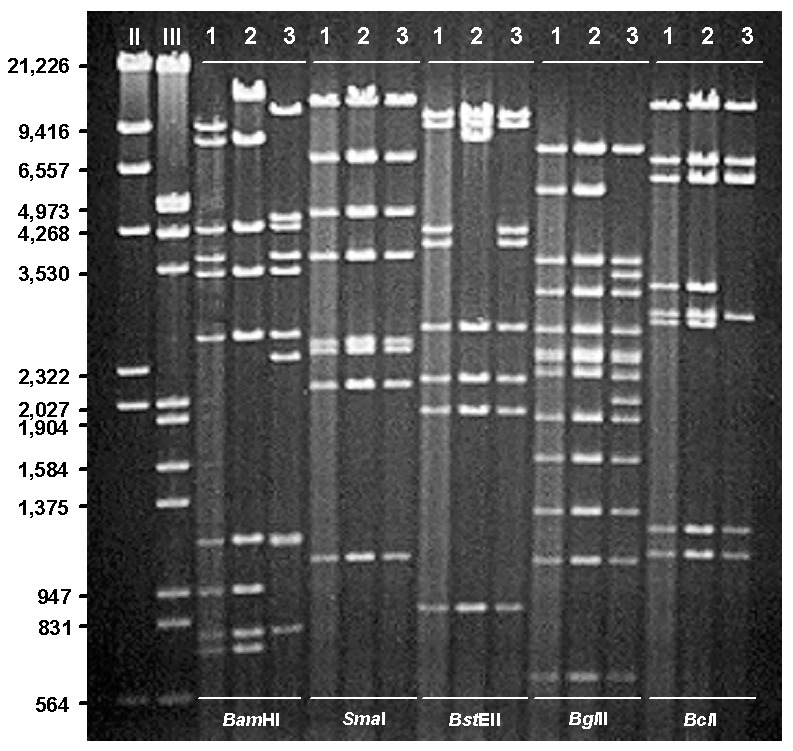
Restriction profiles of representative human adenovirus (Ad) genome types Ad7b (1), Ad7d2 (2), and Ad7h (3) after digestion with selected enzymes, *Bam*HI, Sma I, *Bst*EII, *Bgl*II, and *BcI*I. DNA markers II (λ *Hin*dIII) and III (λ *Hin*dIII/*Eco*RI).

### Ad7 Hexon Gene Sequencing

The hypervariable regions of the hexon gene (corresponding to nucleotides 403 to 1356 of the reference strain Gomen) of 24 temporally and geographically diverse Ad7 field isolates (including 11 Ad7b, 10 Ad7d2, and 3 Ad7h and laboratory strains S-1058, 55142 vaccine, and Gomen) were sequenced and compared with published Ad7 hexon sequences available from GenBank (Table 2). Nucleotide and deduced amino acid alignments of these sequences comprised two major genetic clusters as previously described (*26*,*45*): cluster 1, Ad7p (Gomen) and Ad7p1; and cluster 2, Ad7a Ad7b, Ad7c, Ad7d, Ad7d2, Ad7g, and Ad7h. Cluster 2 sequences were highly conserved, with over 98% nucleotide identity, and were generally uncorrelated with genome type. However, a unique Gln substitution for Leu (codon CTG > CAG) at amino acid position 443 of loop 2 of the predicted hexon protein was identified in all 10 Ad7d2 isolates from the United States and Israel; this substitution was also present in published hexon sequences of Ad7d isolates from China (*45*) and Japan (*38*).

**Table 2:** Human adenovirus 7 (Ad7) field isolates and laboratory strains used for hexon gene sequence comparisons

ID	Genome type	Location	Isolation year	Accession no.	Sequence source^a^
					
S-1058	7a	USA	1955	af053085	Inada & Mukoyama, direct submission; CDC
55142 vaccine	7a3	USA	1963	af065067	Crawford-Miksza et al. (*26*); CDC
BC30	7b	China	1958	u75951	Li & Wadell (*45*)
BC14	7b	China	1965	u77390	Li & Wadell (*45*)
KCH4	7b	England	1973	u77391	Li & Wadell (*45*)
v2026	7b	USA, MI	1986		CDC
v2124	7b	USA, SD	1987		CDC
2000017657	7b	USA, MD	1991		CDC
99026790	7b	USA, OH	1993		CDC
2000017667	7b	USA, MD	1994		CDC
2000026630	7b	USA, NY	1996		CDC
Kn T96-0620	7b	USA, CA	1996	af065068	Crawford-Miksza et al. *(**26*)
99018141	7b	USA, MO	1997		CDC
2000016352	7b	USA, IL	1997		CDC
2000016376	7b	USA, SC	1997		CDC
2000016361	7b	USA, MO	1997		CDC
2000016376	7b	USA, SC	1997		CDC
37300	7c	Sweden	1964	u75952	Li & Wadell, (*45*)
BC3655	7d	China	1981	u77392	Li & Wadell, (*45*)
BC4492	7d	China	1984	u75953	Li & Wadell, (*45*)
BC4609	7d	China	1984	u77393	Li & Wadell (*45*)
BC8488	7d	China	1984	u77394	Li & Wadell (*45*)
383^b^	7d	Japan	1992	af053086	Hashido et al. (*38*)
Bal^b^	7d	Japan	1995	af053087	Hashido et al. (*38*)
2000017663	7d2	USA, MD	1993		CDC
2000026865	7d2	Israel	1993	af321311	CDC
2000017669	7d2	USA, MD	1994		CDC
2000026621	7d2	USA, NY	1995		CDC
99026817	7d2	USA, OH	1995		CDC
2000016333	7d2	USA, IL	1997		CDC
2000016364	7d2	USA, MO	1997		CDC
2000016375	7d2	USA, SC	1997		CDC
98034168	7d2	USA, IL	1998		CDC
2000017983	7d2	USA, WI	1998		CDC
BC25	7g	China	1985	u75954	Li & Wadell (*45*)
87-922	7h	Argentina	1987	u75956	Li & Wadell (*45*)
990179044	7h	Chile	1998		CDC
99018196	7h	USA, AZ	1998		CDC
2000016378	7h	USA, TX	1998		CDC
Gomen	7p	USA	1954	z48571	Li et al., direct submission; CDC
BC3423	7p1	China	1981	u75955	Li & Wadell (*45*)

^a^Sequencing and restriction analysis performed at Centers for Disease Control and Prevention (CDC) or obtained from previously published sources. Published sequences of Ad7 laboratory strains S-1058, Gomen, and 55142 vaccine confirmed at CDC. ^b^Ad7 strains 383 and Bal were originally reported as Ad7d with a "different restriction pattern by *Bst*EII" (*38*).

### Temporal Distribution of Ad7 Genome Types

The yearly distribution of the 166 Ad7 genome types is shown in Table 3. Ad7b was the only genome type identified from 1970 through 1992 and was the predominant genome type identified through 2000. Ad7d2 first appeared among 1993 isolates and accounted for approximately 28% of all Ad7 isolates obtained from 1993 to 2000. Four epidemiologically unrelated isolates of Ad7h were identified in 1998 and 2000.

**Table 3:** Yearly distribution of 166 human adenovirus 7 (Ad7) genome types, United States and Canada, 1966–2000

Genome type	1966-1969	1970-1992	1993	1994	1995	1996	1997^a^	1998^a^	1999	2000	Total

7p	0	0	0	0	0	0	3	0	0	0	3
7a	3	0	0	0	0	0	0	0	0	0	3
7a3	2	0	0	0	0	0	0	0	0	0	2
7b	0	31	6	4	14	8	26	4	6	8	107
7b_var_	0	0	0	0	0	0	0	0	1	0	1
7d2	0	0	2	1	3	4	32	2	0	2	46
7h	0	0	0	0	0	0	0	3	0	1	4
Total	5	31	8	5	17	12	61	9	7	11	166

^a^Data include 50 Ad7 isolates collected in 1997 (47 isolates ) and 1998 (3 isolates ) from military recruit training centers (Table 4).

### Geographic Distribution of Ad7 Genome Types

Ad7b was identified among isolates from nearly all states (and eastern Ontario) sampled. Ad7d2 was first identified in isolates from Maryland and New York in 1993 and thereafter primarily from midwestern and northeastern states, including Wisconsin, Illinois, Kansas, Missouri, Louisiana, South Carolina, and Ohio, as well as eastern Ontario. Ad7h was only identified among isolates obtained from Texas and Arizona.

### Ad7 Civilian Outbreaks, 1996-2000

During this study, we became aware of five separate outbreaks of Ad7 respiratory illness among civilians (Table 4). Four were institutional outbreaks that involved primarily infants and young children with underlying chronic disease that occurred in the fall or summer months of 1996, 1998, 1999, and 2000. A fifth communitywide outbreak of Ad7 in Tennessee, which occurred during March-July 1997, involved previously healthy children (*16*).Genome type analysis at CDC identified Ad7d2 in three of the four outbreaks where isolates were available. We attributed one outbreak to a novel Sma I restriction variant of Ad7b (Ad7b_var_) that occurred in New York in 1999 (Jennifer Calder, manuscript in preparation).

**Table 4:** Recognized civilian outbreaks of human adenovirus 7 (Ad7) respiratory disease, United States, 1996–2000

Location	Date	Setting	No. cases^a^	No. deaths	No. Ad7 isolates	No. restriction	Genome type
Houma, LA^b^	June 1996	Pediatric chronic-care facility	13	7	4	2	7d2

Memphis, TN^c^	Mar 1997	Community acquired	47	1	26	0	nd

Chicago, IL^d^	Nov 1998	Pediatric chronic-care facility	31	8	11	11	7d2
		and tertiary hospital	37	0	6	6	"
							
New York City, NY^e^	Oct 1999	Chronic-care facility for	33	7	15	15	7b_var_
		mentally disabled persons					

Des Moines, IA^f^	Oct 2000	Pediatric chronic-care facility	20	4	9	9	7d2
^a^Suspected and confirmed cases of Ad7 respiratory disease. ^b^Robert Gohd, Children's Hospital, New Oreleans, LA (pers. comm.). ^f^Michael Buley, Iowa Dept of Public Health, Des Moines, IA (pers. comm.). ^c^Mitchell et al. (*16*). ^d^Gerber et al. (*17*). ^e^Jennifer Calder, The Mailman School of Public Health, Columbia University, New York, NY (manuscript in preparation).							

### Ad7 Gnome Types at Military Recruit Training Centers

In anticipation of increased Ad activity following termination of routine vaccination of new military recruits in 1996, the Naval Health Research Center (NHRC) in San Diego, CA, initiated surveillance for new cases of Ad-associated respiratory illness (*20*) [http://www.nhrc.navy.mil/geis/sites/nhrc.htm]. Clinical specimens collected at five designated training centers (San Diego, CA; San Antonio, TX; St. Robert, MO; Great Lakes, IL; and Columbia, SC) from recruits who reported respiratory illness were submitted to NHRC for identification of viral and bacterial pathogens. Of 50 Ad7 isolates, Ad7d2 was the most common genome type identified (58%), followed by Ad7b (34%), Ad7p (6%), and Ad7h (2%) (Table 5). Most Ad7d2 infections were reported from the Naval Recruit Training Center in Great Lakes, IL, where an outbreak of Ad7 (and Ad3) respiratory illness was documented during the fall of 1997 (*21*). Over 70% of the Ad7 isolates sampled from the Great Lakes Center from September 1997 to February 1998 were identified as Ad7d2. One of four Ad7h isolates identified in this study was obtained from a new recruit at Lackland Air Force Base, in San Antonio.

**Table 5:** Genome types of 50 human adenovirus 7 (Ad7) isolates obtained from military recruit training centers, Feb 199–May 1998^a^

	No. Ad	No. Ad	No.	No.	Dates of	Genome type				
Training center	isolates	typed	Ad7 (%)	restriction	isolation	7b	7d2		7p	7h
Marine Corps Recruit Depot, San Diego, CA	129	128	10 (8)	1	April 1997				1	

Lackland Air Force Base, San Antonio, TX	1	1	1 (100)	1	April 1998					1
									
Fort Leonard Wood, St. Robert, MO	266	260	29 (11)	9	Feb 1997 to Nov 1997	4		5		
									
Naval Recruit Training Center, Great Lakes, IL	632	592	396 (67)	28	Sept 1997 to May 1998	8		20		
									
Fort Jackson, Columbia, SC	786	738	66 (9)	11	June 1997 to April 1998	5		4	2	
									
Total	1,814	1,719	502 (29)	50		17		29	3	1

^a^Isolation and serotyping of Ads conducted at the Naval Health Research Center, San Diego, CA.

## Discussion

Our study represents the most comprehensive survey to date of Ad7 genome types circulating in the United States and provides a basis for future surveillance studies that can better delineate the disease impact of these viruses.

Before this study, the most comprehensive surveys of Ad7 genomic variants in the United States were conducted by Wadell et al. (*27*) and Adrian et al. (*28*) with field isolates of Ad7 collected from 1961 to 1985. These authors identified a diverse group of cocirculating Ad7 genome types (Ad7p, Ad7a, Ad7c, and others) that by the late 1960s to early 1970s were replaced by Ad7b, a change that preceded similar shifts to Ad7b seen in other parts of the world. Our data confirm this observation and show a continued dominance of the Ad7b genome type in the United States. Only one genome type from the earlier period, Ad7p, was still identified among currently circulating strains. We also documented the appearance of two new Ad7 genome types: Ad7d2, which was first identified in specimens collected in 1993 and subsequently detected over a wide geographic area in the eastern half of the United States and Canada; and Ad7h, which was first identified in specimens collected in 1998 in the Southwest.

Both epidemiologic and molecular evidence suggests that Ad7d2 entered the United States as part of its recent spread from evolutionarily related Ad7d strains formerly restricted to China. Ad7d2 shows the highest degree of genetic relatedness to Ad7d, differing by only one *Bst*EII restriction site in pairwise comigrating restriction fragment analysis with 12 different endonucleases (*24*,*37*); it possesses the unique amino acid substitution in the hexon protein also present in Ad7d isolates from China (*45*) and Japan (*38*). Ad7d was identified as early as 1980 in Beijing (*24*) and 2 years later in Changchin (*46*), and rapidly displaced Ad7b to become the major genome type circulating in China through 1990. Ad7d was identified in Japan during 1987 to 1992 (*35*) and in Korea in 1995 (18; Hoan-Jong Lee, pers. comm.), and Ad7d2 was the predominant genome type isolated during the 1995-1998 Ad7 epidemic in Japan (*15*,*36*). Ad7d2 was subsequently identified in Israel in 1992 (*37*) and in the United States in this study in 1993.

The emergence and apparent global spread of Ad7d2 are reminiscent of observations for another genome type of serotype 7, Ad7b. Originally described by Wadell and Varsanyi (*25*), Ad7b was associated with outbreaks of severe respiratory illness in Europe in the 1970s (12). Although first isolated in 1956 from a Paris orphanage outbreak (*12*,*47*), subsequent retrospective studies did not identify Ad7b in Europe again until 1969 (*27*). Before then, the earliest documented occurrence of Ad7b was in China in 1958 (*24*), where it was the predominant genome type circulating through the early 1980s (*24*,*46*). With the exception of Paris, the first appearance of Ad7b outside China was on the U.S. West Coast in 1962 (*27*). By 1970, Ad7b was the predominant genome type circulating throughout the United States (*28*) and eventually throughout many parts of the world.

The mechanism(s) underlying the apparent greater fitness of some Ad7 genome types, as reflected by their capacity to displace other circulating strains, remains speculative. Possible explanations include mutations or recombinations that yield strains with increased pathogenicity and therefore greater chance of causing recognized illness, or biological or antigenic changes that enhance transmission or infection compared with other Ad7 genome types. Although there is no conclusive evidence of differences in pathogenicity between Ad7 genome types, some types appear to be more frequently isolated from healthy carriers (e.g., Ad7p and Ad7a), while others are more often isolated from patients with more serious clinical outcomes (e.g., Ad7b, Ad7c, Ad7d, and Ad7h) (*27*,*34*). Some antigenic differences between Ad7 genome types have also been demonstrated; recent studies identified minor differences in neutralization titer between Ad7 prototype strain Gomen (Ad7p) and the vaccine strain 55142 (Ad7a) with rabbit hyperimmune antisera (*26*). In addition, a unique amino acid substitution in the hexon protein that distinguishes Ad7d/Ad7d2 strains from other genome types is predicted to impart substantial changes in the hydrophilicity of the protein and possibly associated antigenic changes (*45*).

Although Ad7 can be spread directly by the respiratory route, efficiency of transmission is typically lower than for some other respiratory viruses. Efficient spread usually requires crowding, such as that in closed communities like chronic-care facilities, military barracks, and day-care centers. Widespread community outbreaks of Ad7 can occur but appear to require low levels of herd immunity. For example, in Japan, >95% of persons <40 years of age lacked specific antibodies to Ad7 before the countrywide epidemic of Ad7 that began in 1995 (*48*,*49*). The most comprehensive recent seroprevalence data on Ad7 in the United States were obtained in 1992 from 364 military basic trainees attending new recruit training centers (*50*). Approximately 73% of screened trainees lacked specific antibodies to Ad7. In another study to evaluate the potential for use of Ad vectors in gene therapy for cystic fibrosis, 73.9% of 46 serum specimens collected from 1993 to 1995 from children (median age 4.7 years) were seronegative for Ad7 (*51*).

To achieve rapid spread, a novel genome type presumably requires an immunologically naive population, greater biological fitness than the indigenous circulating strains, and a means of introduction to the susceptible community. Azar et al. (*37*) noted that the appearance of Ad7d2 in Israel coincided with the arrival of large numbers of immigrants from the former Soviet Union and Ethiopia during the early 1990s. The global spread of Ad7b in the 1960s and 1970s may have been aided by the movement of unvaccinated U.S. and allied military personnel during the Vietnam War. In our study, the appearance of Ad7h in the U.S. Southwest in 1998 may be explained by the emigration of persons from Ad7h-endemic regions of South America, where communitywide outbreaks of respiratory illness due to Ad7h occurred as recently as 1998 in Chile (Rodrigo Fasce, pers. comm.). However, a more comprehensive survey of Ad7 isolates from Mexico and U.S. states on the Mexican Border would be necessary to substantiate this observation.

The five recognized civilian outbreaks of Ad7 respiratory illness that occurred during 1996-2000, three of which we attributed to genome type 7d2, might have been due to increased reporting as a result of our interest in this study or may represent a real increase in Ad7-associated disease, as occurred in Europe during the early 1970s and in Japan and Korea (*12*,*15*,*18*) beginning in 1995. One unsubstantiated possibility is that the discontinuation of vaccination of U.S. military recruits for Ad4 and Ad7 in 1996 and the subsequent increase in Ad-associated disease at military bases throughout the United States (*20*–*22*) provided a new focus for Ad7 dissemination to civilian populations. A possible example of this is the 1998 outbreak of Ad7d2 illness at a Chicago pediatric chronic-care facility described earlier (*17*). This outbreak occurred within a few miles of the Naval Training Center in Great Lakes, which had had an outbreak of Ad7d2 the preceding year (*21*). Most cases of Ad infection at military bases since 1996 have been attributed to Ad4 (*20*,*22*), but no comparable outbreaks of Ad4 disease among civilians have been reported. Unlike Ad7, which poses a risk to both civilian and military populations, Ad4 has only infrequently been associated with outbreaks of respiratory illness in civilian populations (*2*).

Although we identified individual cases of severe lower respiratory tract illness and deaths attributed to Ad7d2 and Ad7h in this study, the possibility that these two genome types may be associated with more severe disease is not yet clear. More extensive clinical and epidemiologic study is required to adequately address this question. The limited data from infected military recruits suggest no differences in clinical illness between those infected with Ad7d2 and Ad7b (data not shown). Reports of Ad7d2 infections in Israel (*37*) and Ad7d infections in China (*46*) also noted no clear differences in severity of disease. Cases of severe pneumonia and neurologic disease were reported from a recent regional epidemic of Ad7d2 in Japan (*15*), but there was no evidence that these severe cases were more common than those reported for outbreaks involving other Ad7 genome types. Ad7h, a genetically unique recombinant between Ad7 and Ad3 (*42*), has been linked to increased illness and death in infants in Chile and Argentina, where it is second only to *Human respiratory syncytial virus* as a cause of severe viral pneumonia in infants and young children (*34*,*52*). However, in this study, too few cases of Ad7h infection were identified to assess differences in disease severity.

In conclusion, our study documents the recent appearance in the United States of two new Ad7 genome types, Ad7d2 and Ad7h, and provides additional evidence of the global spread of these formerly geographically restricted viruses. The possibility that these genome types may be associated with more severe disease makes it prudent to monitor their spread and associated disease.
